# Crystal growth, crystal structure determination, and computational studies of a new mixed (NH_4_)_2_Mn_1–x_Zn_x_(SO_4_)_2_(H_2_O)_6_ Tutton salt

**DOI:** 10.1007/s00894-022-05323-4

**Published:** 2022-10-05

**Authors:** João G. Oliveira Neto, Jailton R. Viana, Jardel B. O. Lopes, Antonio D. S. G. Lima, Marcus L. Sousa, Mateus R. Lage, Stanislav R. Stoyanov, Rossano Lang, Adenilson O. Santos

**Affiliations:** 1 Center for Social Sciences, Health and Technology, Federal University of Maranhão - UFMA, Imperatriz, MA 65900-410 Brazil; 2 Chemistry Institute, State University of Campinas - UNICAMP, Campinas, SP 13083-970 Brazil; 3 Federal University of Maranhão (UFMA), Campus Balsas, MA-140, km 04, Balsas, MA 65800-000 Brazil; 4 Natural Resources Canada, CanmetENERGY Devon, 1 Oil Patch Drive, Devon, AB T9G 1A8 Canada; 5 Institute of Science and Technology, Federal University of São Paulo - UNIFESP, São José dos Campos, SP 12231-280 Brazil

**Keywords:** Crystal growth, Tutton salts, Powder X-ray diffraction, Hirshfeld surface analysis, DFT calculations

## Abstract

Tutton salts have been extensively explored in recent decades due to their attractive physical and chemical properties, which make them potential candidates for thermochemical heat storage systems and optical technologies. In this paper, a series of new mixed Tutton salts with the chemical formula (NH_4_)_2_Mn_1–x_Zn_x_(SO_4_)_2_(H_2_O)_6_ is reported. Crystals are successfully grown by the solvent slow evaporation method and characterized by powder X-ray diffraction (PXRD) with Rietveld refinement. In particular, the crystal structure of the mixed (NH_4_)_2_Mn_0.5_Zn_0.5_(SO_4_)_2_(H_2_O)_6_ crystal is solved through PRXD data using the DICVOL06 algorithm for diffraction pattern indexing and the Le Bail method for lattice parameter and spatial group determination. The structure is refined using the Rietveld method implemented in TOPAS® and reported in the Cambridge Structural Database file number 2104098. Moreover, a computational study using Hirshfeld surface and crystal void analyses is conducted to identify and quantify the intermolecular interactions in the crystal structure as well as to determine the amount of free space in the unit cell. Furthermore, 2D-fingerprint plots are generated to evaluate the main intermolecular contacts that stabilize the crystal lattice. Density functional theory is employed to calculate the structural, thermodynamic, and electronic properties of the coordination [Zn(H_2_O)_6_]^2+^ and [Mn(H_2_O)_6_]^2+^ complexes present in the salts. Molecular orbitals, bond lengths, and the Jahn–Teller effect are also discussed. The findings suggest that in Mn-Zn salts several properties dependent on the electronic structure can be tuned up by modifying the chemical composition.

## Introduction

In recent decades, several inorganic crystalline systems, including Tutton salts, have been widely investigated due to their remarkable physical and chemical properties that direct the possible applications [1–4]. Crystals with optical filter properties are being sought for medical purposes and intelligent vision systems [5]. Thermochemical heat storage materials, which offer an alternative solution to the global energy problem, have also been in a high demand [6, 7]. In this context, Tutton salts stand out due to their versatility in the various branches of science, having the potential to be used as ultraviolet light (UV) filters [8], thermal energy storage materials [9], and other applications [10–14].

Tutton salts form a hexahydrate isomorphic crystallographic family with a general chemical formula M_2_M*(XO_4_)_2_·6H_2_O, where M is occupied by a monocation (Cs^+^, K^+^, Rb^+^, Tl^+^, and NH_4_^+^), M* represents a dication (Mg^2+^, V^2+^, Mn^2+^, Fe^2+^, Co^2+^, Ni^2+^, Cu^2+^, Zn^2+^, and Cd^2+^), and X is a site constituted by S or Se atoms [15, 16]. These salts crystallize in a monoclinic system with P2_1_/*a* space group containing two formulas per unit cell (*Z* = 2) [17]. There are two complexes [M*(H_2_O)_6_] in the unit cell, located in the inversion center and coordinated by six H_2_O molecules in a slightly distorted octahedral environment caused by the Jahn–Teller effect [18]. This distortion occurs mostly in transition metal complexes with octahedral geometry, in which the five *d* atomic orbitals are split into two degenerate systems. If the molecule presents an electronically degenerate ground state, a lower energy state is formed due to a distortion that removes the degeneracy [19].

Hydrogen bonds between the octahedral complexes, [(XO)_4_] tetrahedral groups, and monocations stabilize the crystals [20]. The dications are located at (0, 0, 0) and (½, ½, 0) positions in the unit cell, while the other atoms are in general positions [21]. Besides the Tutton salts formed from individual compounds in the dication site, there are also mixed Tutton salts that crystallize with two hetero-metallic dications in the M* site of the crystal. The latter may exhibit unique properties due to the combination of characteristics of the precursor compounds [22, 23].

Several works report the synthesis of mixed Tutton salt crystals and their structural and optical properties, such as K_2_Zn_x_Ni_1–x_(SO_4_)_2_·6H_2_O [24], K_2_Co_x_Ni_1-x_(SO_4_)_2_·6H_2_O [10], (NH_4_)_2_Zn_x_Mg_1-x_(SO_4_)_2_·6H_2_O [17], (NH_4_)_2_Ni_x_Cu_1-x_(SO_4_)_2_·6H_2_O [25], and (NH_4_)_2_Ni_x_Co_1-x_(SO_4_)_2_·6H_2_O [12]. However, few papers provide studies involving the occupation of Mn^2+^ and Zn^2+^ cations in the lattice structure of an individual or mixed Tutton salt [15, 26, 27]. Furthermore, few reports present computational investigations with information about bonding interactions in these systems [28]. It is known that computational chemistry tools, such as Hirshfeld surfaces, crystal voids, and density functional theory (DFT) calculations, can provide valuable features in support of experimental data for a better understanding of the interactions of constituent species in a Tutton salt, as well as their properties [24, 26].

In this work, the successful synthesis of new mixed Tutton salts named (NH_4_)_2_Mn_1-x_Zn_x_(SO_4_)_2_(H_2_O)_6_ is presented. The crystal structures were solved by powder X-ray diffraction (PXRD) assisted by the Rietveld refinement method. The effects of inserting Mn^2+^ and Zn^2+^ ions in the lattice structure were also analyzed. Moreover, Hirshfeld surface and crystal voids were generated to comprehend the intermolecular interactions in the crystals. DFT calculations were also performed to assess the structural, thermodynamic, and electronic properties of the main coordination compounds present in the salts.

## Experimental procedures

### Crystal growth

Tutton salts were synthesized by the slow evaporation method from the saturated solution at a constant temperature of 308 K, using deionized water as a solvent and the following precursor reagents: ammonium sulfate ((NH_4_)_2_SO_4_, Vetec, 99%), manganese sulfate monohydrate (MnSO_4_(H_2_O), Vetec, 98%), and zinc sulfate heptahydrate (ZnSO_4_(H_2_O)_7_, Impex, 99%). For the synthesis of the series, the (NH_4_)_2_SO_4_ mass was fixed at 5.0 g, and the MnSO_4_(H_2_O) and ZnSO_4_(H_2_O)_7_ masses were calculated from the stoichiometric ratio of the chemical equation (Eq. 1):

1 $${\left({\mathrm{NH}}_{4}\right)_{2}\mathrm{SO}}_{4}+{\mathrm{MnSO}}_{4}\left({\mathrm{H}}_{2}\mathrm{O}\right)+{\mathrm{ZnSO}}_{4}\left({\mathrm{H}}_{2}\mathrm{O}\right)_{7}\to {\left({\mathrm{NH}}_{4}\right)}_{2}{\mathrm{Mn}}_{1-\mathrm{x}}{\mathrm{Zn}}_{\mathrm{x}}{\left({\mathrm{SO}}_{4}\right)}_{2}{\left({\mathrm{H}}_{2}\mathrm{O}\right)}_{6}+\uparrow {2\mathrm{H}}_{2}\mathrm{O}$$

The mole ratios and reagent quantities used in the syntheses are shown in Table 1. To obtain the mixed crystals labeled NMn_1-x_Zn_x_SOH, the mole ratios of the transition metals (Mn and Zn) were set to *x* = 0.0, 0.3, 0.5, 0.7, and 1.0. The masses of the starting compounds were homogenized with approximately 50 mL of deionized water. The solutions were heated to ≈ 348 K and stirred at 460 RPM using a magnetic stirrer for 5 h. The solutions pH will be given later (Table 2). The final solutions were filtered to remove impurities, covered with parafilm, and left in a temperature-stabilized oven (308 K) for solid-phase nucleation.

**Table 1:** Molar ratios and amounts of (NH_4_)_2_SO_4_, MnSO_4_(H_2_O), and ZnSO_4_(H_2_O)_7_ used in the solution preparations

(NH_4_)_2_SO_4_ [g]	*x*	MnSO_4_(H_2_O) [g]	1–*x*	ZnSO_4_(H_2_O)_7_ [g]	NMn_1–x_Zn_x_SOH
5.000	1.00	8.451	0.00	0.000	Mn_1.0_Zn_0.0_
5.000	0.70	5.915	0.30	4.313	Mn_0.7_Zn_0.3_
5.000	0.50	4.225	0.50	7.169	Mn_0.5_Zn_0.5_
5.000	0.30	2.535	0.70	10.064	Mn_0.3_Zn_0.7_
5.000	0.00	0.000	1.00	14.378	Mn_0.0_Zn_1.0_

**Table 2:** Chemical and physical data of solutions and crystals

NMn_1–x_Zn_x_SOH	Solution conductivity [mV]	pH	Growth time [days]	Morphology	Dimensions LxWxH [cm^3^]	Color
Mn_1.0_Zn_0.0_	191.3	3.52	18	SC	0.81 × 1.32 × 0.38	LP
Mn_0.7_Zn_0.3_	203.3	3.33	21	SC	0.98 × 1.47 × 0.56	LP
Mn_0.5_Zn_0.5_	194.5	3.50	21	SC	0.70 × 1.22 × 0.25	W
Mn_0.3_Zn_0.7_	180.1	3.73	21	PC	1.04 × 1.59 × 0.60	YW
Mn_0.0_Zn_1.0_	138.6	4.33	18	PC	1.24 × 1.97 × 0.67	YW

*SC* single crystal, *PC* polycrystal, *LP* light pink, *W* white, *YW* yellowish white

### Structural characterization

The PXRD patterns at room temperature were collected using an Empyrean powder diffractometer (PANalytical), with CuKα radiation (*λ* = 1.5418 Å) and operating at 40 kV/40 mA. The diffractograms were recorded in the 2*θ* angular range between 10 and 50°, with an angular step of 0.02° and an acquisition time of 2 s. Rietveld refinement method [29] using the GSAS/EXPGUI software [30] was applied to the experimental diffractograms with previously resolved structures.

However, for the (NH_4_)_2_Mn_0.5_Zn_0.5_(SO_4_)_2_(H_2_O)_6_ sample, the crystal structure was solved using the algorithm DICVOL06 [31] implemented in the software Expo2014 [32] to perform the diffraction pattern indexing. Afterwards, the Le Bail method [33] implemented in the GSAS/EXPGUI software was used to extract the reflection intensities. In a complementary way, the atomic positions were obtained by taking advantage of the coordinates in the crystallographic information (.cif) file 43310 [34] accessed from the Inorganic Crystal Structure Database (ICSD). Finally, the Rietveld method implemented in TOPAS® software version 4.2 was applied to refine the PXRD pattern until it reached suitable parameters. The final.cif file was checked by using the enCIFer software version 1.7.5. The supplementary crystallographic data for the structure NMn_0.5_Zn_0.5_SOH were inputted in CSD file number 2104098. In addition, the calculated pattern with the structure packing was prepared and analyzed by Mercury software version 4.3.1.

### Hirshfeld surface, 2D-fingerprint plots, and unit cell voids

Hirshfeld surfaces, 2D-fingerprint plots, and unit cell voids were generated using the Crystal Explorer 17 software [35] to thoroughly analyze the intermolecular interactions between the chemical species and evaluate the occupied volume in the unit cell. The calculated 3D Hirshfeld surfaces were mapped as a function of the normalized distance (*d*_norm_), displaying a standard color scheme, where the most distant contacts were represented by blue color, contacts close to the van der Waals radius were shown in white, and the close contacts were indicated in red color [36, 37]. The 2D-fingerprint graphs were presented as a function of the distance from a given point on the Hirshfeld surface to the closest atom outside the surface (*d*_*e*_) and the distance from a given point on the Hirshfeld surface to the closest atom inside the surface (*d*_*i*_). The 2D-fingerprint plots encompassed all intermolecular contacts, quantifying specific interactions and summarizing the contributions of each spatial region and functional group to intermolecular interactions [38]. Void spaces were identified through isosurfaces of 0.002 a.u. of procrystal electron density, as suggested by Bader et al. [39].

### Computational studies

The geometry optimization calculations were performed by employing the DFT functional PBE1PBE [40] in the gas phase. The hybrid PBE1PBE functional was selected based on reports that it produces geometric and spectroscopic properties in a very good agreement with experimental results, in particular, for first row transition metal complexes with high and low spin states [41, 42]. The core shells of Mn and Zn were treated with the QZV effective core potentials, while the quadruple-ζ QZVP basis set was used for valence shell electrons. The H and O atom shells were considered entirely using the quadruple-ζ QZVP basis set [43].

The ground state of [Mn(H_2_O)_6_)]^2+^, a *d*^*5*^ complex, had a spin multiplicity equal to 6. The ground state was determined as the state with a lower energy after full geometry optimization between the quartet and sextet states with spin multiplicities of 4 and 6, respectively. The spin multiplicity is defined as *s*(*s* + 1), where *s* equals the number of unpaired electrons times the electronic spin value of ½.

The ground state spin multiplicity of [Zn(H_2_O)_6_)]^2+^, a *d*^10^ complex, was equal to 1. Each ground state–optimized geometry was confirmed to be a minimum in the potential energy surface using vibrational frequency calculations. The DFT calculations were carried out with the software Gaussian 16 [44]. The electronic singlet states were treated using the restricted PBE1PBE functional, whereas the states with higher multiplicities were treated using unrestricted PBE1PBE functional.

The results of the electronic structure calculations were analyzed using the ChemCraft 1.8 software [45]. Additionally, the electrostatic potential values were calculated using the Multiwfn software package [46]. These values were useful for the study of intermolecular interactions between the species in the crystal structure.

## Results and discussion

### Growth of NMn_1–x_Zn_x_SOH Tutton salts

**Fig. 1:**
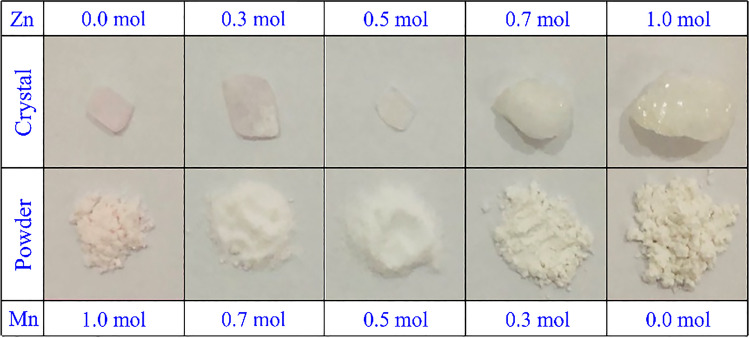
NMn_1–x_Zn_x_SOH samples grown by the slow evaporation technique with different mole ratios between Mn and Zn. Images of as-grown and pulverized (powder form) crystals

The NMn_1–x_Zn_x_SOH crystals have been successfully obtained by the slow evaporation method in an acid medium for a period ranging from 18 to 21 days. As can be observed in Fig. 1, the samples with low Zn concentrations (up to 50%) are grown as single crystals with well-defined faces. The good optical transparency and smooth surface of the NMn_0.5_Zn_0.5_SOH crystal are noticeable to the naked eye. In contrast, a polycrystalline growth pattern is seen for samples with concentrations greater than 50% Zn. It was also verified that the coloring changed from light pink to slightly yellowish white, according to the Mn concentration reduction. The crystal dimensions significantly varied in maximum size. Table 2 exhibits a summary of the chemical and physical data for the NMn_1–x_Zn_x_SOH Tutton salts.

### PXRD and structure determination

**Fig. 2:**
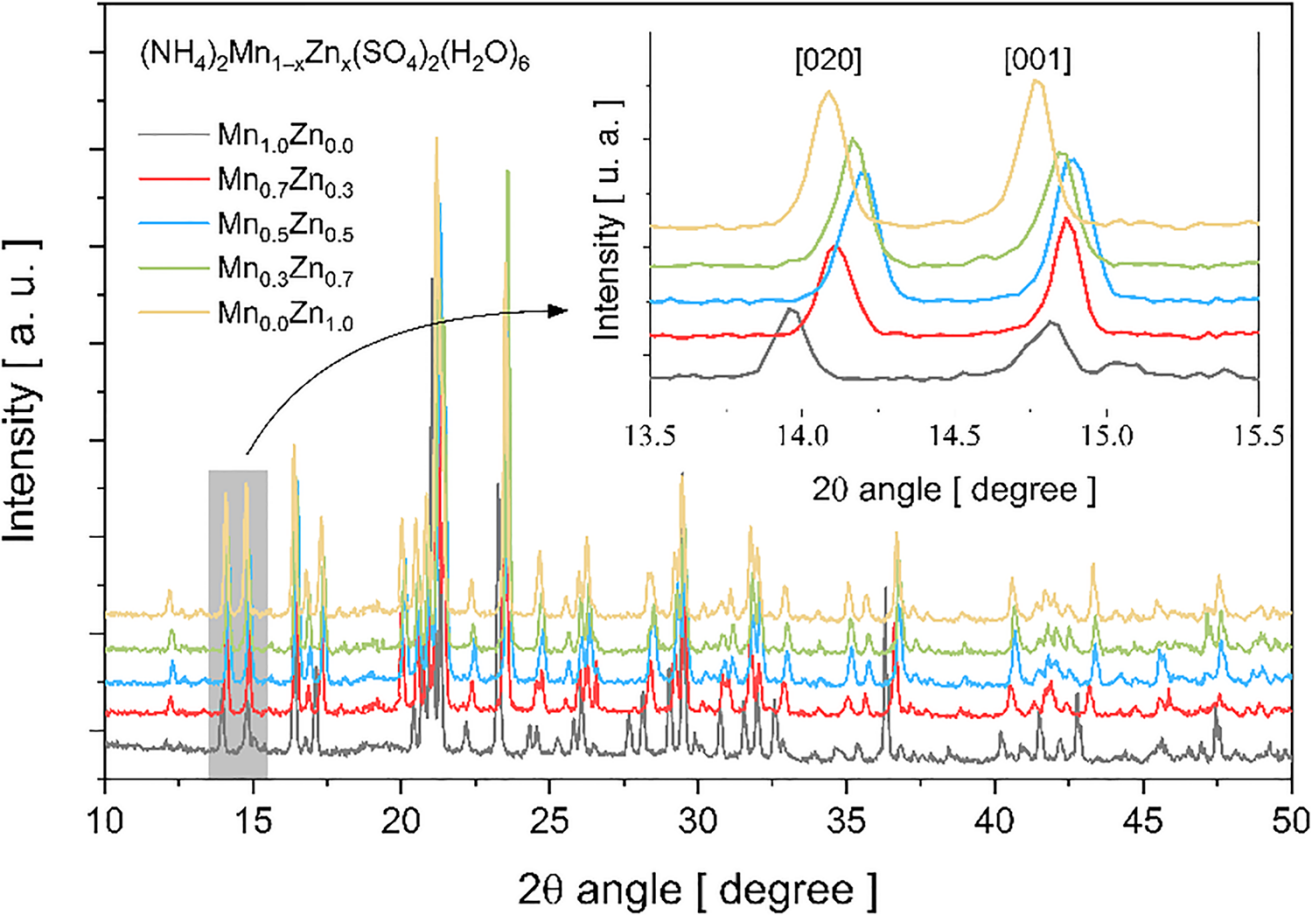
PXRD patterns of NMn_1–x_Zn_x_SOH crystals in the angular range between 10 and 50°. Inset: an example of observed shifts in diffraction peaks (2*θ* interval between 13.5 and 15.5°)

Figure 2 shows the PXRD patterns obtained at room temperature for the series of NMn_1–x_Zn_x_SOH samples. Compared with the NMn_1.0_Zn_0.0_SOH and NMn_0.0_Zn_1.0_SOH reference compounds, the diffraction peaks of the mixed crystals are shifted to larger angles, as observed in the inset for [020] and [001] crystalline planes, for instance. Emphasis is placed on the NMn_0.5_Zn_0.5_SOH sample that presents the most significant structural variation. Chemical composition changes in the lattice structure cause pressure effects at the M* site, arising from the atomic radii difference between Mn (2.05 Å) and Zn (2.01 Å).

The PXRD patterns of the individual (NH_4_)_2_Mn(SO_4_)_2_(H_2_O)_6_ and (NH_4_)_2_Zn(SO_4_)_2_(H_2_O)_6_ crystals are analyzed by the Rietveld method using the structures already resolved and found in the ICSD database at file numbers [14378] and [16591], respectively. For the refinement of the mixed crystal, the second divalent ion is inserted, and the occupation factor corresponding to the number of moles added in the NMn_1–x_Zn_x_SOH is modified. However, to solve the NMn_0.5_Zn_0.5_SOH structure accurately, it has been necessary to use distinct mathematical algorithms, as described in the “Structural characterization” section, due to considerable structural changes observed in the diffractogram (Fig. 2).

**Fig. 3:**
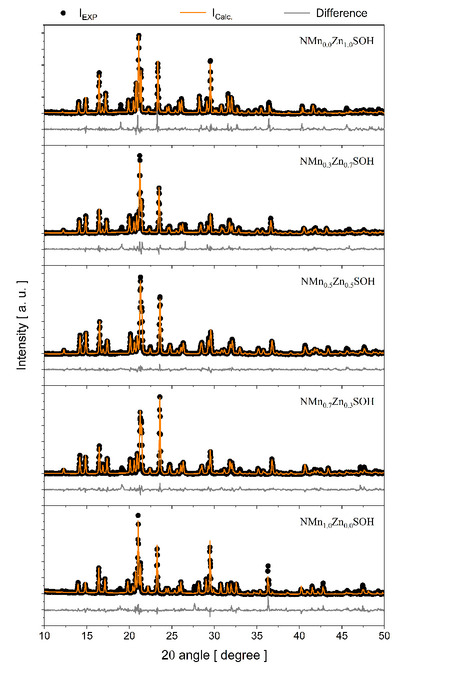
PXRD patterns refined by the Rietveld method of the NMn_1–x_Zn_x_SOH series. Experimental data are indicated by open circles and the calculated profile as the continuous red lines overlying them. The gray bottom continuous curves represent the difference between the calculated and experimental intensities

Figure 3 displays the refined PXRD patterns. All samples crystallize in monoclinic symmetry (P2_1_/*a* space group); each unit cell contains two formulas (*Z* = 2). Therefore, crystals belong to the isomorphic crystallographic family of Tutton salts. Table 3 summarizes the lattice, structural, and refinement quality parameters provided by the Rietveld refinements. The data indicate that the insertion of two divalent hetero-metallic species at the same site causes a decrease in the unit cell volume due to the reduction of *a* and *b* lattice parameters. Again, such behavior is expected since the atomic radius of Zn is smaller than that of Mn. Thus, the addition of Zn^2+^ ions in the NMn_1-x_Zn_x_SOH framework, with the respective non-occupation of the site by Mn^2+^ ions, induces a contraction in the structural packaging of mixed crystals. The *c* parameter does not change as much as *a* and *b*. Regarding the statistical parameters of refinement, the indicators (*R*_wp_, *R*_p_, and *S*) show good reliability of the procedures.

**Table 3:** Unit cell parameters calculated by the Rietveld refinement method of the NMn_1–x_Zn_x_SOH crystal series

		NMn_1–x_Zn_x_SOH				
		Mn_1.0_Zn_0.0_	Mn_0.7_Zn_0.3_	Mn_0.5_Zn_0.5_	Mn_0.3_Zn_0.7_	Mn_0.0_Zn_1.0_
Lattice parameters	*a* [Å]	9.371 (5)	9.278 (5)	9.256 (12)	9.271 (5)	9.343 (6)
	*b* [Å]	12.687 (6)	12.560 (6)	12.540 (17)	12.559 (5)	12.645 (8)
	*c* [Å]	6.246 (9)	6.234 (4)	6.245 (81)	6.236 (3)	6.239 (9)
	α = γ [°]	90.00	90.00	90.00	90.00	90.00
	β [°]	106.899 (5)	106.94 (6)	106.88 (35)	106.93 (4)	106.92 (5)
	V [Å^3^]	710.67 (4)	694.98 (5)	693.73 (16)	694.13 (7)	705.35 (5)
Structural parameters	Symmetry	Monoclinic	Monoclinic	Monoclinic	Monoclinic	Monoclinic
	SG	P2_1_/*a*	P2_1_/*a*	P2_1_/*a*	P2_1_/*a*	P2_1_/*a*
	*Z*	2	2	2	2	2
Refinement quality parameters	*R*_wp_ [%]	9.51	9.47	8.24	9.73	9.76
	*R*_p_ [%]	7.36	7.25	6.36	7.14	7.73
	*S*	1.86	1.94	1.45	1.95	2.28

*SG* space group, *R*_*wp*_ weighted profile *R*-factor, *R*_*p*_ profile *R*-factor, *S* goodness of fit

**Fig. 4:**
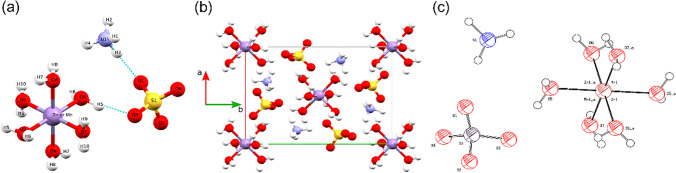
Mixed NMn_0.5_Zn_0.5_SOH crystal. **a** Molecular structure. **b** Molecular packing. **c** ORTEP diagram of the solved structure at the 50% probability level for the thermal ellipsoids

The asymmetric unit and the unit cell projection on *ab* planes of the solved NMn_0.5_Zn_0.5_SOH structure are presented in Fig. 4. The NMn_0.5_Zn_0.5_SOH unit (Fig. 4a) consists of a [M*(H_2_O)_6_] metal complex with a slightly distorted octahedral structure, a [SO_4_]^2–^ group with a tetrahedral arrangement linked to the metal complex via (O5-H5···O3) hydrogen bonds, and an NH_4_^+^ unit with a tetrahedral structure bonded to the [SO_4_]^2–^ group by (N1-H3···O1) bonds.

The NMn_0.5_Zn_0.5_SOH unit cell (Fig. 4b) has an atomic ordering of packaging in octahedral (containing [M*(H_2_O)_6_] moiety) and tetrahedral (containing [SO_4_]^2–^ and NH_4_^+^ moieties) layers. The Mn–O and Zn–O bond lengths present three distinct values: 2.141(26) Å, 2.094(28) Å, and 2.011(28) Å (see Table 4). The O–Mn–O and O–Zn–O bond angles range from 92.50(11)° to 87.90(99)°. These differences confirm that the octahedral geometry of the metal complex is slightly distorted due to the Jahn–Teller effect. An ORTEP diagram with atom numbering is shown in Fig. 4c.

**Table 4:** Geometric parameters obtained from the solved NMn_0.5_Z_0.5_SOH structure

Bond length [Å]		Bond angle [°]	
Zn–Mn	0.000	O5–Mn/Zn–O6	87.90 (99)
Mn/Zn–O5	2.141 (26)	O5–Mn/Zn–O7	89.6 (10)
Mn/Zn–O6	2.094 (28)	O6–Mn/Zn–O7	92.5 (11)
Mn/Zn–O7	2.011 (28)	H5–O5–Mn/Zn	104 (15)
O5–H5	1.01 (27)	H6–O5–Mn/Zn	119 (19)
O5–H6	0.83 (32)	H7–O6–Mn/Zn	114 (24)
O6–H7	0.85 (35)	H8–O6–Mn/Zn	109 (20)
O6–H8	0.91 (39)	H9–O7–Mn/Zn	109 (12)
O7–H9	1.05 (28)	H10–O7–Mn/Zn	127 (22)
O7–H10	0.83 (26)	H5–O5–H6	125 (23)
S–H5	2.81 (23)	H7–O6–H8	112 (24)
O3–H5	1.92 (26)	H9–O7–H10	101 (26)
S–O1	1.479 (29)	S–O3–H5	109.0 (82)
S–O2	1.388 (45)	O1–S–O2	104.4 (19)
S–O3	1.510 (27)	O2–S–O3	99.6 (18)
S–O4	1.525 (35)	O3–S–O4	114.3 (19)
N–O1	3.046 (29)	O4–S–O1	110.9 (17)
H3–O1	2.052 (19)	N–H3–O1	164.6 (14)
N–H1	1.020 (20)	H1–N–H2	105.3 (20)
N–H2	1.083 (25)	H2–N–H3	102.5 (15)
N–H3	1.032 (17)	H3–N–H4	114.7 (22)
N–H4	0.934 (19)	H4–N–H1	115.9 (19)

The oxygen atoms of [SO_4_]^2–^ groups in NMn_0.5_Zn_0.5_SOH crystal act as hydrogen bond acceptors of the type O–H···O, where the H_2_O molecules in the [M*(H_2_O)_6_] metal complex participate as hydrogen bond donors; and of the type N–H···O, where NH_4_ molecules also participate as H donors. All these bonds are listed in Table 5, along with the distance [Å] between the hydrogen bond donor and acceptor atoms.

**Table 5:** Main hydrogen bonding interactions estimated of the solved NMn_0.5_Z_0.5_SOH structure, considering contacts shorter than the sum of van der Waals radii

Molecules	Atomic dimers	Distance [Å]			Angle [°]
		H ···Acceptor	Donor ··· H	Donor ···Acceptor	
Mn/Zn(OH_2_)_6_ and SO_4_	O5···H5···O3	1.925	1.010	2.924	169.8
	O5···H6···O4	2.014	0.827	2.834	171.3
	O6···H7···O2	1.957	0.854	2.806	172.8
	O6···H8···O4	1.840	0.899	2.728	168.7
	O7···H9···O3	1.911	1.052	2.925	160.5
	O7···H10···O1	1.841	0.832	2.656	166.3
NH_4_ and SO_4_	N1···H1···O1	2.079	1.021	3.047	157.6
	N1···H1···O2	2.395	1.021	3.261	142.0
	N1···H2···O3	1.560	1.083	2.638	173.0
	N1···H3···O1	2.052	1.032	3.059	164.6
	N1···H4···O4	2.008	0.933	2.791	140.4

### Hirshfeld surface and 2D-fingerprint plot analysis

**Fig. 5:**
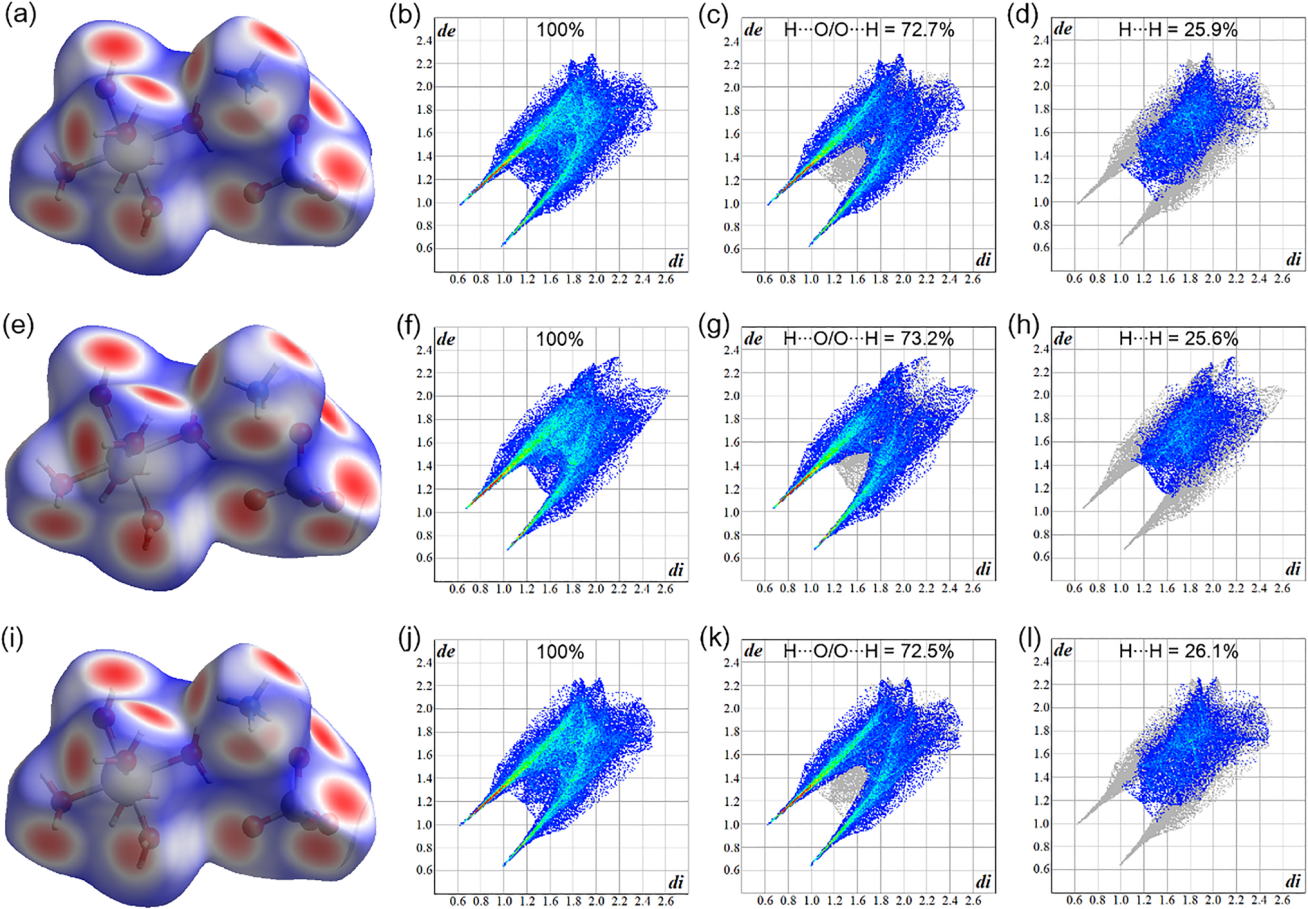
Hirshfeld surface mapping according to *d*_norm_: **a** NMn_1.0_Zn_0.0_SOH, **e** NMn_0.0_Zn_1.0_SOH, and **i** NMn_0.5_Zn_0.5_SOH. Full 2D-fingerprint plots: **b** NMn_1.0_Zn_0.0_SOH, **f** NMn_0.0_Zn_1.0_SOH, and **j** NMn_0.5_Zn_0.5_SOH. H···O/O···H interactions 2D-fingerprint plots: **c** NMn_1.0_Zn_0.0_SOH, **g** NMn_0.0_Zn_1.0_SOH, and **k** NMn_0.5_Zn_0.5_SOH. H···H interactions 2D-fingerprint plots: **d** NMn_1.0_Zn_0.0_SOH, **h** NMn_0.0_Zn_1.0_SOH, and **l** NMn_0.5_Zn_0.5_SOH

For a better understanding of intermolecular interactions, Hirshfeld surfaces and 2D-fingerprint plots have been obtained and examined. The molecular unit occupation in the crystal lattice is determined, as shown in Fig. 5, to divide the electron density of the crystal into molecular fragments. Figure 5a, e, and i illustrate Hirshfeld surfaces of NMn_1.0_Zn_0.0_SOH, NMn_0.0_Zn_1.0_SOH, and NMn_0.5_Zn_0.5_SOH salts, respectively, plotted as the normalized distance *d*_norm_. The color coding describes the intensity of the intermolecular contacts: red regions are intermolecular contacts with distances shorter than the van der Waals radii, white regions are intermolecular contacts with distances equal to van der Waals radii, and blue regions are intermolecular contacts with distances longer than the van der Waals radii. In the three structures, the red regions indicate stronger interactions around the hydroxyl group, with a predominance of H···O/O···H contacts. Slightly red and white regions associated with H···H and O···O contacts on Hirshfeld surfaces are also observed.

Hirshfeld surfaces are associated with 2D-fingerprint plots, where it is possible to observe a histogram as a function of the *d*_*e*_ and *d*_*i*_ properties that represent a fraction of colored points on the surface, in which the red points account for specific close contacts and the blue points for distant contacts. This calculation used in this tool provides a quantitative analysis of the different types of intermolecular interactions occurring in a crystal, which enables the analysis of attraction and repulsion interactions among the species constituting a crystal.

The cumulative 2D-fingerprint plots are shown in Fig. 5b, f, and j, referring to NMn_1.0_Zn_0.0_SOH, NMn_0.0_Zn_1.0_SOH, and NMn_0.5_Zn_0.5_SOH Tutton salts, respectively. The similarity noticed in these graphs occurs because these materials belong to the same crystallographic isomorphic family, as determined by PXRD technique. The decomposed graphs of the H···O/O···H and H···H interactions with corresponding percentage contributions are shown in Fig. 5c, d, g, h, k, and l.

It is observed for the three crystals that the H···O/O···H interactions contribute to more than 70.0% of the Hirshfeld surface, which is the main contact stabilizing the NMn_1–x_Zn_x_SOH crystal lattices. This can also be confirmed by the presence of high, sharp red peaks in the regions of low values of *d*_*e*_ and *d*_*i*_ (see in Fig. 5c, g, and k), which indicates the occurrence of strong interactions.

The H···H interactions are also accounted for in the packaging of these crystals, totaling an average of 25.9% of these contacts (see Fig. 5d, h, and l). Weak dispersive forces of the O···O type are also quantified, however with values below 1.4%. Therefore, it can be stated that NH_4_^+^, [SO_4_]^2–^, and M*(H_2_O)_6_ are co-crystallized in the structure mainly influenced by H···O/O···H and H···H contacts. Moreover, the variation of Mn^2+^ and Zn^2+^ content at the M*(H_2_O)_6_ bivalent site causes slight structural changes compared to NMn_1.0_Zn_0.0_SOH and NMn_0.0_Zn_1.0_SOH crystals. In addition to the PXRD results, these variations are also observed in the 2D-fingerprint of this sample, where it is possible to observe that the occupation of different metals in the NMn_0.5_Zn_0.5_SOH structure causes a small reduction in the percentage of H···O/O···H contacts and a slight increase in H···H contacts. Possibly this occurs due to the pressure effect promoted between the two metal centers in the unit cell.

### Crystal voids

**Fig. 6:**
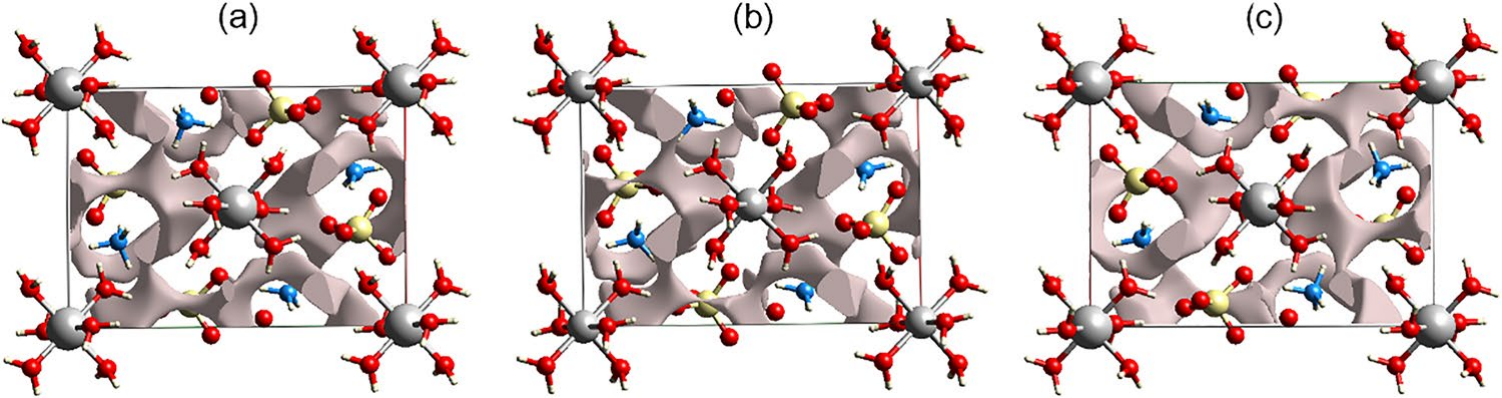
Crystal voids (viewed along the *b*-axis in the unit cell) of the samples. **a** NMn_1.0_Zn_0.0_SOH. **b** NMn_0.0_Zn_1.0_SOH. **c** NMn_0.5_Zn_0.5_SOH

Figure 6a–c depict the void spaces in the unit cells of the NMn_1.0_Zn_0.0_SOH, NMn_0.0_Zn_1.0_SOH, and NMn_0.5_Zn_0.5_SOH crystals. The voids are visualized through electronic density isosurfaces, where it is possible to calculate the free volume of the unit cell, the surface area, and the percentage of voids present in the crystalline solids.

According to computational results, the NMn_1.0_Zn_0.0_SOH sample (Fig. 5a) presents a void volume of 52.81 Å^3^, corresponding to 7.43% of void spaces in the unit cell and a void surface area of 221.19 Å^2^. However, the NMn_0.0_Zn_1.0_SOH sample (Fig. 5b) exhibits higher values, with a percentage of 8.53% of void spaces associated with a volume of 59.98 Å^3^, and a void surface area of 239.09 Å^2^. This change can be explained by the atomic radii of transition metals since both samples have the same molecular structure and differ only in the coordination sphere. As the atomic radius of Mn is greater by 0.04 Å than that of Zn, the former takes up more space in the unit cell and decreases the void volume.

The NMn_0.5_Zn_0.5_SOH sample presents intermediate values when compared to the others, with 8.01% of void space in the unit cell, void volume of 55.45 Å^3^, and void surface area of 225.91 Å^2^. It is also observed that in the three samples, the isosurfaces are not completely closed around all the molecules but are open in the regions where interactions among functional groups occur. Furthermore, the presence of a high percentage of void spaces can cause a decrease in the energy of the interactions and, consequently, reduce the lattice energy, affecting physicochemical parameters, such as solubility, dissolution, and hardness of crystal structures. It is important to emphasize that these crystal voids, Hirshfeld surfaces, and 2D-fingerprint plots show structural properties and intermolecular interactions that have not previously been reported in the literature for NMn_1–x_Zn_x_SOH Tutton salts.

### Coordination complexes study

The Mn(II) and Zn(II) metal complexes contain six H_2_O molecules in the octahedral structures of the NMn_1.0_Zn_0.0_SOH and NMn_0.0_Zn_1.0_SOH salts, and have been computationally investigated using DFT. The geometry optimization results reveal that the sextet ground state of [Mn(H_2_O)_6_]^2+^ is more stable than the quartet state by 48.8 kcal/mol and 48.4 kcal/mol in terms of the Gibbs free energy and total electronic energy with zero-point vibrational energy (ZPVE) correction, respectively. The uncoordinated Mn^2+^ also has a sextet ground state. The energy of coordination is analyzed in terms of the variations of Gibbs free energy (Δ*G*^298^), enthalpy (Δ*H*), and electronic energy, corrected with ZPVE in vacuum (Δ*E*_ZPVE_), in accordance with Eqs. (2–4):

2 $${\Delta }_{\mathrm{coord}}{G}^{298}={\Delta G}^{298}{\left[\mathrm{M}{\left({\mathrm{H}}_{2}\mathrm{O}\right)}_{6}\right]}^{2+}-\left(6\Delta {G}^{298}\left[{\mathrm{H}}_{2}\mathrm{O}\right]+\Delta {G}^{298}\left[{\mathrm{M}}^{2+}\right]\right)$$

3 $${\Delta }_{\mathrm{coord}}H=\Delta H{\left[\mathrm{M}{\left({\mathrm{H}}_{2}\mathrm{O}\right)}_{6}\right]}^{2+}-\left(6\Delta H\left[{\mathrm{H}}_{2}\mathrm{O}\right]+\Delta H\left[{\mathrm{M}}^{2+}\right]\right)$$

4 $${\Delta }_{\mathrm{coord}}{E}_{\mathrm{ZPVE}}={\Delta E}_{\mathrm{ZPVE}}{\left[\mathrm{M}{\left({\mathrm{H}}_{2}\mathrm{O}\right)}_{6}\right]}^{2+}-\left(6\Delta {E}_{\mathrm{ZPVE}}\left[{\mathrm{H}}_{2}\mathrm{O}\right]+\Delta {E}_{\mathrm{ZPVE}}\left[{\mathrm{M}}^{2+}\right]\right)$$

The Δ*G*^298^, Δ*H*, and Δ*E*_ZPVE_ results are listed in Table 6. The ZPVE-corrected coordination electronic energies (Δ_coord_*E*_ZPVE_) of [Zn(H_2_O)_6_]^2+^ and the sextet ground state of [Mn(H_2_O)_6_]^2+^ are equal to − 334.1 kcal/mol and − 293.2 kcal/mol, respectively. Additionally, the Δ_coord_*E*_ZPVE_ of [Mn(H_2_O)_6_]^2+^ optimized in the quartet state is − 326.0 kcal/mol. These results indicate that the zinc(II) aqua-complex is more stable in comparison to the manganese(II) aqua-complex. The thermochemical Gibbs free energy and enthalpy values show the same tendency as the total electronic energy. The thermodynamic parameters of all complexes investigated in this study are shown in Tables 6 and 7.

**Table 6:** Coordination Gibbs free energy (Δ_coord_*G*^298^), enthalpy (Δ_coord_*H*), and ZPVE-corrected electronic energy (Δ_coord_*E*_ZPVE_) of the complexes in kcal/mol

System	Δ_coord_*G*^298^	Δ_coord_*H*	Δ_coord_*E*_ZPVE_
[Mn(H_2_O)_6_]^2+^ quartet	–274.9	–329.5	–326.0
[Mn(H_2_O)_6_]^2+^ sextet	–242.2	–296.6	–293.2
[Zn(H_2_O)_6_]^2+^	–282.2	–337.8	–334.1

**Table 7:** Selected calculated geometric and electronic properties of [Zn(H_2_O)_6_]^2+^, [Mn(H_2_O)_6_]^2+^ sextet, and [Mn(H_2_O)_6_]^2+^ quartet. q(O) = Mulliken charge of O atom from H_2_O; *ρ*_*s*_(O) = spin density of O atom from H_2_O

Complex	M–O bond [Å]	*q*(O)	*ρ*_*s*_(O)
[Zn(H_2_O)_6_]^2+^	2.10	–0.40	-
[Mn(H_2_O)_6_]^2+^ sextet	2.20	–0.39	0.01
[Mn(H_2_O)_6_]^2+^ quartet	2.02	–0.36	0.00
[Mn(H_2_O)_6_]^2+^ quartet	2.10	–0.39	0.01
[Mn(H_2_O)_6_]^2+^ quartet	2.26	–0.39	0.01

**Fig. 7:**
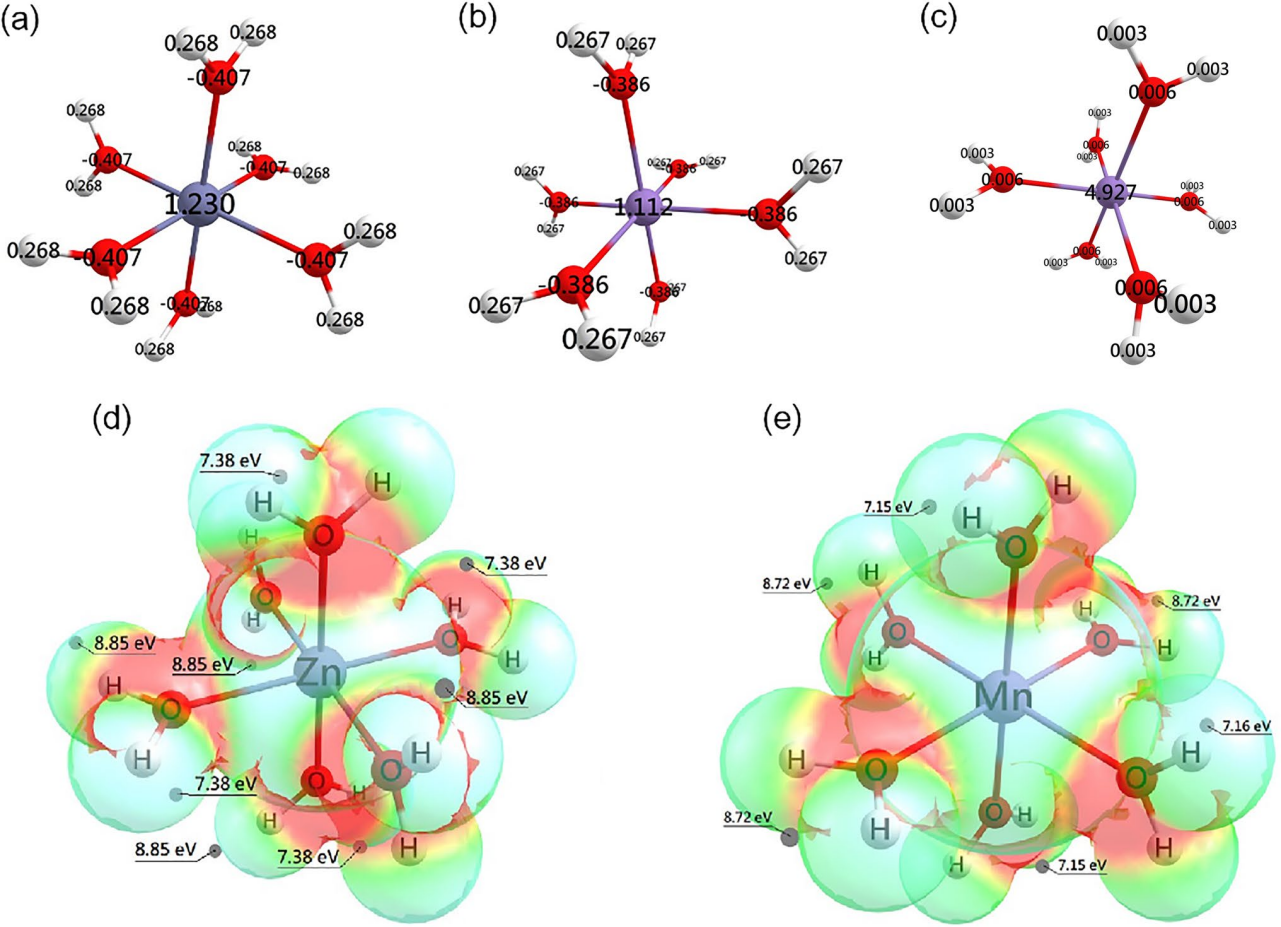
Partial atomic charges of aqua-complex hexacoordinated of **a** [Zn(H_2_O)_6_]^2+^ and **b** [Mn(H_2_O)_6_]^2+^ sextet, **c** with the spin density of [Mn(H_2_O)_6_]^2+^sextet. Electrostatic potentials of **d** [Zn(H_2_O)_6_]^2+^ and **e** [Mn(H_2_O)_6_]^2+^ sextet mapped on the electron density surface, and selected values

The Mulliken atomic charges calculated for both metal complexes yield similar values for the atoms of the H_2_O molecules, being symmetrically distributed for all of them, but reveal significant differences for the metal ions. The electronic properties of the zinc and manganese hexaaqua complexes in their ground states are presented in Fig. 7. The Mulliken charge values of 1.230 |*e*| and 1.112 |*e*| are calculated for the metal centers zinc and manganese, respectively, as shown in Fig. 7a and b, highlighting a significant depletion of electronic charge from the metal dications to H_2_O molecules, being more pronounced for the manganese than the zinc complex. The Mulliken atomic spin values of [Mn(H_2_O)_6_]^2+^ in the sextet ground state (Fig. 7c) show that the most spin density, attributed to unpaired electron distribution, is localized on the Mn atom, equal to 4.93 relative to α electrons.

The electrostatic potential surfaces (EPS) of the complexes are shown in Fig. 7d and e. The EPS are represented in a color gradient, varying from red color for regions with negative partial charges to blue color for regions with positive partial charges. In the same images, the values of electrostatic potentials calculated are shown, in eV, in specific sites of the complexes. Higher and lower electrostatic potential values are mainly localized near the hydrogen and oxygen atoms, respectively, of H_2_O ligands. This representation provides insight into how each moiety contributes to the salt formation process.

It is important to note that the results presented in Fig. 7 indicate that the [Zn(H_2_O)_6_]^2+^ and [Mn(H_2_O)_6_]^2+^ complexes are octahedral and do not exhibit the Jahn–Teller effect distortion found in the crystal structure, in particular the Mn–O bond lengths (Table 4). To help elucidate this distortion, the quartet state of [Mn(H_2_O)_6_]^2+^ with an energy 48.4 kcal/mol higher than that of the sextet ground state is considered. In Table 7, we summarize the geometrical and electronic properties of [Zn(H_2_O)_6_]^2+^ and [Mn(H_2_O)_6_]^2+^ sextet along with those of the [Mn(H_2_O)_6_]^2+^ quartet. The results show that the [Mn(H_2_O)_6_]^2+^ quartet features three distinct M–O bond lengths that, in terms of bond alternation, are analogous to the Mn–O bond lengths (Mn–O5, Mn–O6, and Mn–O7) listed in Table 4. These three bond lengths also correlate with three values of the O atom charges and indicate that the pairs of H_2_O molecules *trans* with respect to Mn are equivalent by symmetry. This Jahn–Teller effect reflected in Mn–O bond lengths and O atom charges and spins could stabilize the quartet state of [Mn(H_2_O)_6_]^2+^ in the conditions of the crystal structure.

**Fig. 8:**
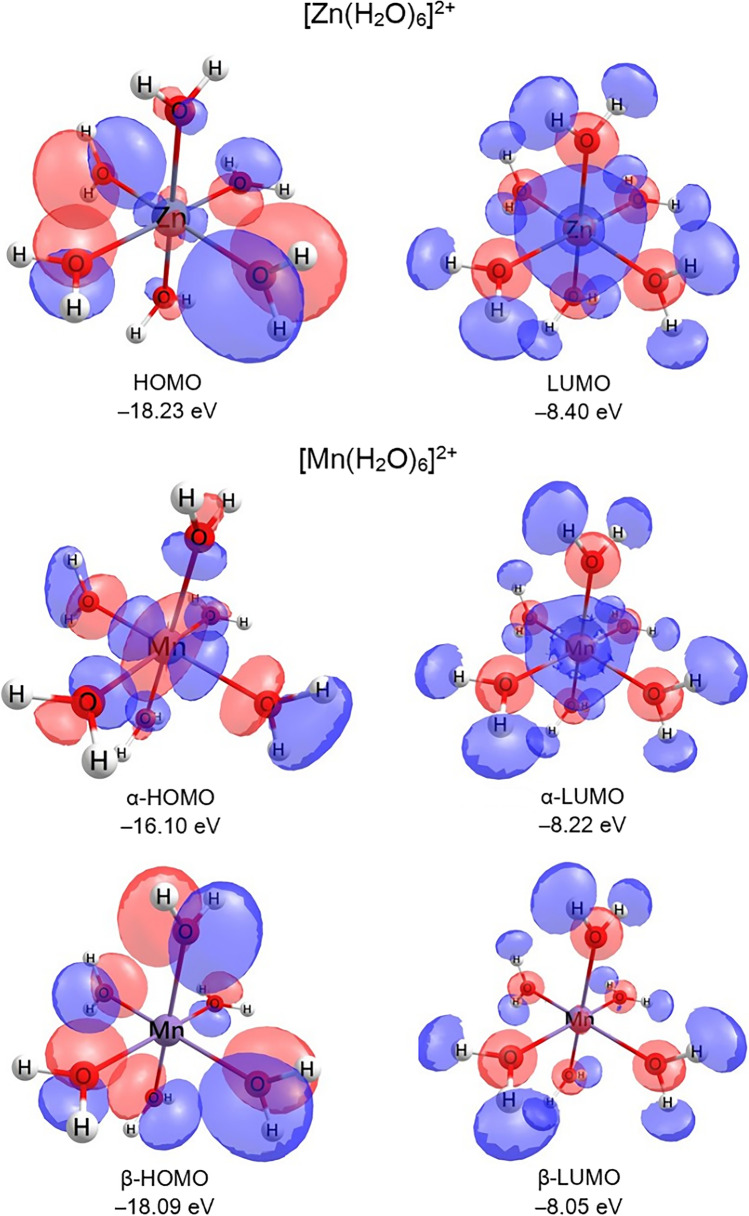
Molecular orbitals HOMO and LUMO of [Zn(H_2_O)_6_]^2+^ singlet as well as α-HOMO and -LUMO, and β-HOMO and -LUMO of [Mn(H_2_O)_6_]^2+^ sextet

The highest occupied molecular orbitals (HOMO) and lowest unoccupied molecular orbitals (LUMO) are also calculated. The [Zn(H_2_O)_6_]^2+^ complex corresponds to a closed-shell system with a direct energy gap (Δ*E*_HOMO-LUMO_) of 9.83 eV between a HOMO with paired electrons and the LUMO. Since the [Mn(H_2_O)_6_]^2+^ sextet complex is treated with double determinants, the Δ*E*_HOMO-LUMO_ is defined in terms of *alfa* (α) and *beta* (β) orbitals. The true HOMO is α-HOMO, as it has higher energy than the β-HOMO, and therefore, the Δ*E*_HOMO-LUMO_ is 7.89 eV. The spatial distributions of the HOMO and LUMO of the complexes are shown in Fig. 8. The lowest energy transition of the [Zn(H_2_O)_6_]^2+^ involves a charge transfer from two H_2_O ligands to the metal and can be assigned as a ligand-to-metal charge transfer. The lowest energy transitions of the [Mn(H_2_O)_6_]^2+^ sextet are metal-centered, based mainly on α-HOMO to α-LUMO spatial distributions. The α-HOMO of [Mn(H_2_O)_6_]^2+^ features a characteristic *d*_z_^2^ spatial distribution.

## Conclusions

In this work, three new crystals of mixed NMn_1–x_Zn_x_SOH Tutton salts (*x* = 0.3, 0.5, and 0.7) were successfully grown by the solvent slow evaporation method using supersaturated solutions. The samples were characterized by PXRD, where it was possible to infer that Mn^2+^ and Zn^2+^ cations in the same lattice site promoted a pressure effect on the crystal structure, leading to the formation of a distorted octahedron resulting from the Jahn–Teller effect. The NMn_0.5_Zn_0.5_SOH crystal had its structure solved due to the changes observed in the PXRD pattern when compared to the other salts. Nonetheless, Rietveld refinement indicated that all crystals exhibit monoclinic symmetry with P2_1_/*a* space group. The structure of NMn_0.5_Zn_0.5_SOH was reported in CSD 2104098.

Moreover, a computational investigation was carried out using Hirshfeld surface and crystal void space analyses, contributing to the intermolecular interactions study and determination of free spaces in the unit cells. Through the Hirshfeld surfaces, the main interaction contacts in the structures were identified. These contacts were quantified using 2D-fingerprint plots, where it was possible to estimate the frequency of occurrence of different intermolecular interactions. The void spaces in unit cells were visualized by isosurfaces, and the unit cell free volume, surface area, and percentage of voids in the crystalline solids were computed.

The DFT studies were conducted to investigate the electronic properties of the aqua-complexes present in the structure of the salts. Mulliken partial atomic charges, electrostatic potential maps, frontier molecular orbitals, and Δ*E*_HOMO-LUMO_ were determined. These results indicated that both the manganese(II) and zinc(II) aqua-complexes were electronically stable, in accordance with the calculated Δ*E*_HOMO-LUMO_ values and thermochemistry results listed in Table 6. The quartet state of [Mn(H_2_O)_6_]^2+^ featured a geometrical distortion that was correlated with the Mn–O bond alternation in the crystal structure, which was attributed to the Jahn–Teller effect. Furthermore, the results also suggested that the high multiplicity of the manganese(II) aqua-complex and the resultant closely spaced α and β molecular orbitals provided many possibilities for electronic transitions. The closely spaced α-(HOMO and LUMO) and β-(HOMO and LUMO) suggested that different electronic transitions would occur in [Mn(H_2_O)_6_]^2+^ than in [Zn(H_2_O)_6_]^2+^ complex.

These new NMn_1–x_Zn_x_SOH Tutton salts feature attractive crystal, electronic, and bonding properties. Such characteristics and properties suggested an outstanding potential as smart materials sensitive to ultraviolet radiation.

## Data Availability

Data and materials are available on request from the authors.

## Code availability

This work was conducted using the commercial software Gaussian 16, ChemCraft, and the freeware Crystal Explorer 17.

## References

[1] Barpanda P, Oyama G, Ling CD (2014). Krohnkite-type Na_2_Fe(SO_4_)_2_·2H_2_O as a novel 3.25 V insertion compound for Na-ion batteries. Chem Mater.

[2] Marinova D, Kostov V, Nikolova R (2015). From kröhnkite-to alluaudite-type of structure: novel method of synthesis of sodium manganese sulfates with electrochemical properties in alkali-metal ion batteries. J Mater Chem A.

[3] Wang X, Zhuang X, Su G (2008). A new ultraviolet filter: Rb_2_Ni (SO_4_)_2_·6H_2_O (RNSH) single crystal. Opt Mater.

[4] Oliveira Neto JG, Lang R, Rodrigues JAO (2022). Kröhnkite-type K_2_Mn(SO_4_)_2_(H_2_O)_2_ double salt: synthesis, structure, and properties. J Mater Sci.

[5] Vasil’eva NA, Sorokina NI, Antipin AM (2015). Transformation of the structure in a series of mixed K_2_Ni_x_Co_1-x_(SO_4_)_2_·6H_2_O single crystals. JETP Lett.

[6] Sharma A, Tyagi VV, Chen CR (2009). Review on thermal energy storage with phase change materials and applications. Renew Sustain Energy Rev.

[7] Donker’s PAJ, Sögütoglu LC, Huinink HP (2017). A review of salt hydrates for seasonal heat storage in domestic applications. Appl Energy.

[8] Tutton AEH (1901). A comparative crystallographical study of the double selenates of the series R_2_M(SeO_4_)_2_, 6H_2_O. -salts in which M is zinc. Proc R Soc Lond.

[9] Ousaleh HA, Sair S, Zaki A (2020). Advanced experimental investigation of double hydrated salts and their composite for improved cycling stability and metal compatibility for long-term heat storage technologies. Renew Energy.

[10] Manomenova VL, Rudneva EB, Voloshin AE (2016). Crystals of the simple and complex nickel and cobalt sulfates as optical filters for the solar-blind technology. Russ Chem Rev.

[11] Souemti A, Lozano-Gorrín AD, Zayani L (2016). Synthesis, characterization and electrical properties of both pure and cobalt-doped picromerite-type hydrated double salt K_2_Mg_1−x_Co_x_(SO_4_)_2_·6H_2_O (x = 0, 0.4). J Electron Mater.

[12] Ghosh S, Oliveira M, Pacheco TS (2018). Growth and characterization of ammonium nickel-cobalt sulfate Tutton’s salt for UV light applications. J Cryst Growth.

[13] Ganesh G, Ramadoss A, Kannan PS, Subbiahpandi A (2013). Crystal growth, structural, thermal, and dielectric characterization of Tutton salt (NH_4_)_2_Fe(SO_4_)_2_·6H_2_O crystals. J Therm Anal Calorim.

[14] Souamti A, Martín IR, Zayani L (2017). Luminescence properties of Pr^3+^ ion doped Mg-picromerite Tutton salt. J Lumin.

[15] Abu El-Fadl A, Nashaat AM (2017). Growth, structural, and spectral characterizations of potassium and ammonium zinc sulfate hydrate single crystals. Appl Phys A Mater Sci Process.

[16] Pacheco TS, Ghosh S, de Oliveira M, Barbosa AA, Perpétuo GJ, Franco CJ (2017). Growth and characterization of potassium cobalt nickel sulfatehexahydrate crystals: a new UV light filter. J Sci Adv Mater Devices.

[17] Ramasamy G, Bhagavannarayana G, Madhurambal G, Meenakshisundaram S (2012). Crystal growth, structure, crystalline perfection and characterization of zinc magnesium ammonium sulfate hexahydrate mixed crystals Zn_x_Mg_(1–x)_(NH_4_)_2_(SO_4_)_2_·6H_2_O. J Cryst Growth.

[18] Colaneri MJ, Vitali J (2021). Effect of the lattice field on the electronic structure and dynamics of copper − hexahydrate in Tutton salts. J Phys Chem A.

[19] Colaneri MJ, Teat SJ, Vitali J (2020). Electron paramagnetic resonance characteristics and crystal structure of a Tutton salt analogue: copper-doped cadmium creatininium sulfate. J Phys Chem A.

[20] Bejaoui A, Souamti A, Kahlaoui M (2019). Synthesis, characterization, thermal analysis and electrical properties of (NH_4_)_2_M(SO_4_)_2_·6H_2_O (M = Cu Co, Ni). Mater Sci Eng B Solid-State Mater Adv Technol.

[21] Lim AR (2012). Thermodynamic properties and phase transitions of Tutton salt (NH_4_)_2_Co(SO_4_)_2_·6H_2_O crystals. J Therm Anal Calorim.

[22] Parthiban S, Anandalakshmi H, Senthilkumar S (2012). Influence of Vo(II) doping on the thermal and optical properties of magnesium rubidium sulfate hexahydrate crystals. J Therm Anal Calorim.

[23] Vasilyeva NA, Baskakova SS, Lyasnikova MS (2019). Growth and study of properties of mixed crystals (NH_4_)_2_Ni_x_Co_1-__x_(SO_4_)_2_·6H_2_O. Crystallogr Rep.

[24] Zhuang X, Su G, Wang G (2004). Structure, growth and optical properties of Zn_0.24_Ni_0.76_(SO_4_)·7H_2_O single crystal. Cryst Res Technol.

[25] Ghosh S, Lima AH, Flôres LS (2018). Growth and characterization of ammonium nickel-copper sulfate hexahydrate: a new crystal of Tutton’s salt family for the application in solar-blind technology. Opt Mater.

[26] Pacheco TS, Ludwig ZMC, Ullah S (2021). Magnetic characterization, electronic structure and vibrational properties of (NH_4_)_2_M(SO_4_)_2_·6H_2_O (M=Mn, Ni) crystals. Solid State Commun.

[27] Vijila Manonmoni J, Bhagavannarayana G, Ramasamy G (2014). Growth, structure and spectral studies of a novel mixed crystal potassium zinc manganese sulphate. Spectrochim Acta A Mol Biomol Spectrosc.

[28] Barashkov MV, Komyak AI, N SS,  (2000). Vibrational spectra and crystal lattice dynamics of hexahydrates of zinc potassium and ammonium sulfates. J Appl Spectrosc.

[29] Rietveld HM (1967). Line profiles of neutron powder-diffraction peaks for structure refinement. Acta Crystallogr.

[30] Toby BH (2001). EXPGUI, a graphical user interface for GSAS. J Appl Crystallogr.

[31] Boultif A, Louër D (1991). Indexing of powder diffraction patterns for low-symmetry lattices by the successive dichotomy method. J Appl Crystallogr.

[32] Altomare A, Cuocci C, Giacovazzo C, Moliterni A, Rizzi R, Corriero N, Falcicchio A (2013). EXPO2013: a kit of tools for phasing crystal structures from powder data. J Appl Crystallogr.

[33] Le BA, Duroy H, FourQuet JL (1988). Ab-initio structure determination of LiSbW08 by X-ray powder diffraction. Mater Res Bull.

[34] Montgomery H, Lingafelter EC (1964). The crystal structure of Tutton’s salts. II. Magnesium ammonium sulfate hexahydrate and nickel ammonium sulfate hexahydrate. Acta Crystallogr.

[35] Spackman PR, Turner MJ, McKinnon JJ, Wolff SK, Grimwood DJ, Jayatilaka D, Spackman MA (2021). CrystalExplorer: a program for Hirshfeld surface analysis, visualization and quantitative analysis of molecular crystals. J Appl Crystallogr.

[36] McKinnon JJ, Spackman MA, Mitchell AS (2004). Novel tools for visualizing and exploring intermolecular interactions in molecular crystals. Acta Crystallogr B: Struct Sci Cryst Eng Mater.

[37] Spackman MA, Jayatilaka D (2009). Hirshfeld surface analysis. CrystEngComm.

[38] Tan SL, Jotani MM, Tiekink ERT (2019) Utilizing Hirshfeld surface calculations, non-covalent interaction (NCI) plots and the calculation of interaction energies in the analysis of molecular packing. Acta Crystallogr Sect E Crystallogr Commun:308-318. 10.1107/S2056989019001129 10.1107/S2056989019001129 PMC6399703 30867939

[39] Bader RFW, Henneker WH, Cade PE (1967). Molecular charge distributions and chemical binding. J Chem Phys.

[40] Adamo C, Barone V (1999). Toward reliable density functional methods without adjustable parameters: The PBE0 model. J Chem Phys.

[41] Mortensen SR, Kepp KP (2015). Spin propensities of octahedral complexes from density functional theory. J Phys Chem A.

[42] Spillebout F, Stoyanov SR, Zelyak O, Stryker JM, Kovalenko A (2022). Computational investigation of the metal and ligand substitution effects on the structure and electronic states of the phosphoranimide tetramer complexes of Cu(I), Ni(I), Co(I), and Fe(I). Inorg Chem.

[43] Weigend F (2006). Accurate Coulomb-fitting basis sets for H to Rn. Phys Chem Chem Phys.

[44] Frisch MJ, Trucks GW, Schlegel HB (2016). Gaussian 16, Rev. B.01.

[45] Zhurko GA, Zhurko DA (2005) Chemcraft-graphical program for visualization of quantum chemistry computations. Ivanovo, Russia. http://www.chemcraftprog.com

[46] Lu T, Chen F (2012). Multiwfn: a multifunctional wavefunction analyzer. J Comput Chem.

